# Metagenome-Based Metabolic Reconstruction Reveals the Ecophysiological Function of *Epsilonproteobacteria* in a Hydrocarbon-Contaminated Sulfidic Aquifer

**DOI:** 10.3389/fmicb.2015.01396

**Published:** 2015-12-10

**Authors:** Andreas H. Keller, Kathleen M. Schleinitz, Robert Starke, Stefan Bertilsson, Carsten Vogt, Sabine Kleinsteuber

**Affiliations:** ^1^Department of Isotope Biogeochemistry, Helmholtz Centre for Environmental Research – UFZLeipzig, Germany; ^2^Department of Environmental Microbiology, Helmholtz Centre for Environmental Research – UFZLeipzig, Germany; ^3^Department of Proteomics, Helmholtz Centre for Environmental Research – UFZLeipzig, Germany; ^4^Department of Ecology and Genetics, Limnology and Science for Life Laboratory, Uppsala UniversityUppsala, Sweden

**Keywords:** *Campylobacterales*, sulfur cycling, rTCA cycle, acetate assimilation, anaerobic hydrocarbon degradation, nitrogen fixation, niche adaptation, intermediary ecosystem metabolism

## Abstract

The population genome of an uncultured bacterium assigned to the *Campylobacterales* (*Epsilonproteobacteria*) was reconstructed from a metagenome dataset obtained by whole-genome shotgun pyrosequencing. Genomic DNA was extracted from a sulfate-reducing, *m*-xylene-mineralizing enrichment culture isolated from groundwater of a benzene-contaminated sulfidic aquifer. The identical epsilonproteobacterial phylotype has previously been detected in toluene- or benzene-mineralizing, sulfate-reducing consortia enriched from the same site. Previous stable isotope probing (SIP) experiments with ^13^C_6_-labeled benzene suggested that this phylotype assimilates benzene-derived carbon in a syntrophic benzene-mineralizing consortium that uses sulfate as terminal electron acceptor. However, the type of energy metabolism and the ecophysiological function of this epsilonproteobacterium within aromatic hydrocarbon-degrading consortia and in the sulfidic aquifer are poorly understood. Annotation of the epsilonproteobacterial population genome suggests that the bacterium plays a key role in sulfur cycling as indicated by the presence of an *sqr* gene encoding a sulfide quinone oxidoreductase and *psr* genes encoding a polysulfide reductase. It may gain energy by using sulfide or hydrogen/formate as electron donors. Polysulfide, fumarate, as well as oxygen are potential electron acceptors. Auto- or mixotrophic carbon metabolism seems plausible since a complete reductive citric acid cycle was detected. Thus the bacterium can thrive in pristine groundwater as well as in hydrocarbon-contaminated aquifers. In hydrocarbon-contaminated sulfidic habitats, the epsilonproteobacterium may generate energy by coupling the oxidation of hydrogen or formate and highly abundant sulfide with the reduction of fumarate and/or polysulfide, accompanied by efficient assimilation of acetate produced during fermentation or incomplete oxidation of hydrocarbons. The highly efficient assimilation of acetate was recently demonstrated by a pulsed ^13^C_2_-acetate protein SIP experiment. The capability of nitrogen fixation as indicated by the presence of *nif* genes may provide a selective advantage in nitrogen-depleted habitats. Based on this metabolic reconstruction, we propose acetate capture and sulfur cycling as key functions of *Epsilonproteobacteria* within the intermediary ecosystem metabolism of hydrocarbon-rich sulfidic sediments.

## Introduction

Representatives of the *Epsilonproteobacteria* inhabit a broad spectrum of environments like mammalian digestive systems (Wolin et al., 1961; Engberg et al., 2000), brackish water (Brettar et al., 2006), hydrothermal sediments (Inagaki et al., 2003), or subsurface systems (Watanabe et al., 2000; Gittel et al., 2012; Hubert et al., 2012; Handley et al., 2013). Previously obtained isolates have been described as chemolithoautotrophs (Takai et al., 2006; Sievert et al., 2008) fixing carbon via the reductive tricarboxylic acid (rTCA) cycle (Hügler et al., 2005). Notably, they are recognized as key players in sulfidic habitats (Campbell et al., 2006) capable of oxidizing sulfide, sulfur or thiosulfate, or using elemental sulfur/polysulfide as terminal electron acceptors. Furthermore, oxygen, nitrate, and fumarate can be electron acceptors (Campbell et al., 2006; Miller et al., 2007). Besides reduced inorganic sulfur compounds, hydrogen, or organic substances such as malate and formate were shown to be electron donors (Campbell et al., 2006). The broad spectrum of habitats where *Epsilonproteobacteria* can be found is underlined by their capability to grow under aerobic, microaerobic, or anoxic conditions. A model organism representing the metabolic versatility of this proteobacterial class is *Wolinella succinogenes*. It couples anaerobic fumarate or nitrate respiration with hydrogen, sulfide, or formate oxidation (Kröger et al., 2002; Kern and Simon, 2009) but can also grow under limited oxic conditions (Wolin et al., 1961). During the last decade, research focused on *Epsilonproteobacteria* thriving in marine systems such as hydrothermal vents (Miroshnichenko et al., 2004; Nakagawa et al., 2007; Schauer et al., 2011; Stokke et al., 2015) or pelagic oxic-anoxic interfaces (Grote et al., 2012) as well as in terrestrial sulfidic caves and springs (Engel et al., 2004; Porter and Engel, 2008; Jones et al., 2010; Rossmassler et al., 2012; Hamilton et al., 2015), or mud volcanos (Green-Saxena et al., 2012) where they are thought to be mainly involved in the oxidation or reduction of sulfur compounds.

Recently, *Epsilonproteobacteria* were also found to be abundant in anoxic hydrocarbon-rich habitats like oil reservoirs (Hubert et al., 2012), phenol-degrading methanogenic sludge (Zhangh et al., 2005), petroleum-contaminated soil (Kasai et al., 2005), and hydrocarbon-degrading sulfate-reducing enrichment cultures (Kleinsteuber et al., 2008; Müller et al., 2009; Jehmlich et al., 2010; Pilloni et al., 2011; Bozinovski et al., 2012; Kuppardt et al., 2014). The metabolism of *Epsilonproteobacteria* in anoxic hydrocarbon-contaminated subsurface systems and especially in sulfate-reducing consortia is poorly understood as they are neither known to perform dissimilatory sulfate reduction nor to degrade aromatic or aliphatic hydrocarbons. However, they seem to be stimulated by acetate amendment in anaerobic sediments (Handley et al., 2013) or even assimilate acetate as shown by DNA stable isotope probing (SIP) with ^13^C_2_-labeled acetate (Webster et al., 2010).

In this study, we investigated a member of the epsilonproteobacterial order *Campylobacterales* originating from a sulfidic, hydrocarbon-contaminated aquifer at an industrial site near Zeitz, Germany (Schirmer et al., 2006; Vogt et al., 2007; Herrmann et al., 2008). It was originally enriched under sulfate-reducing conditions in a syntrophic, benzene-mineralizing consortium and was shown to be distantly related to the genus *Sulfurovum* (Kleinsteuber et al., 2008; Herrmann et al., 2010). An identical phylotype (in the following named as Zeitz epsilonproteobacterium) was consistently detected in various toluene-degrading (Müller et al., 2009; Jehmlich et al., 2010; Kuppardt et al., 2014) and *m*-xylene-degrading (Bozinovski et al., 2012) sulfate-reducing enrichment cultures from the same site and remained abundant after prolonged incubation. The closest relatives of this phylotype based on 16S rRNA gene sequences were found in pristine sulfidic springs and caves (Porter and Engel, 2008; Jones et al., 2010). A DNA-SIP experiment with ^13^C_6_-benzene revealed significant labeling of the Zeitz epsilonproteobacterium, besides the putative initial benzene degrader, a clostridial phylotype assigned to the genus *Pelotomaculum* (Herrmann et al., 2010).

However, the respective protein-SIP experiment did not confirm labeling of the Zeitz epsilonproteobacterium whereas benzene assimilation by the initial degrader *Pelotomaculum* sp. was verified (Taubert et al., 2012). Likewise, protein-SIP with methyl-labeled *m*-xylene (1,3-dimethyl-^13^C_2_-benzene) did not lead to a labeling of epsilonproteobacterial peptides within the *m-*xylene-degrading enrichment culture (Bozinovski et al., 2012, 2014). Thus, the Zeitz epsilonproteobacterium seems to be not primarily involved in hydrocarbon degradation, despite being consistently present in the respective consortia in varying relative abundances. We hypothesize that it uses metabolites from hydrocarbon degradation under sulfate-reducing conditions, but the type of energy metabolism and its specific ecophysiological role in the consortia have remained unknown so far. To shed light on the ecological niche and metabolic function of *Epsilonproteobacteria* in hydrocarbon-rich sulfidic environments, we aimed at a metabolic reconstruction of the Zeitz epsilonproteobacterium based on genome-centric metagenomics.

## Materials and Methods

### DNA Isolation and Whole Genome Amplification

Cells originated from a batch culture used as a control in a growth experiment with an *m*-xylene-degrading, sulfate-reducing batch culture (Herrmann et al., 2009; Bozinovski et al., 2012, 2014). The medium in the control culture did not contain any organic carbon source. During the experiment, phylogenetic composition was determined by terminal restriction fragment length polymorphism (T-RFLP) analysis using the restriction enzymes *Bst*UI and *Rsa*I according to methods described previously (Ziganshin et al., 2011). It revealed an exceptionally high proportion of the epsilonproteobacterial terminal restriction fragment (T-RF) in the control culture. Cells from 20 mL of this control culture were harvested by centrifugation. DNA was extracted using the NucleoSpin Tissue Kit (Macherey-Nagel) according to the manufacturer’s support protocol for bacteria. Multiple displacement amplification (MDA) of the extracted DNA was performed with the illustra GenomiPhi V2 Amplification Kit (GE Healthcare Life Sciences). Five parallel MDA reactions were carried out according to the manufacturer’s instructions, using a reaction time of 2 h. Phylogenetic composition of each MDA product was determined by T-RFLP analysis as stated above. The relative abundance of the epsilonproteobacterial T-RF was estimated to be 85.5–87.6% (*Bst*UI) and 92.9–94.7% (*Rsa*I), respectively. The reaction products were purified using the Amicon Ultra-0.5 Centrifugal Filter Device (Millipore). DNA quantity and quality was checked photometrically using a NanoDrop ND-1000 UV/Vis spectral photometer (PeqLab, Germany) and by agarose gel electrophoresis. The products of the five MDA reactions were then pooled and used for whole genome sequencing.

### Whole Genome Shotgun Sequencing and Sequence Analysis

Amplified DNA was sequenced in two separate runs with 200 cycles on the Roche 454 FLX platform using Titanium chemistry. The average read length of the first run (310 Mbp) was 391 bp. The second run (235 Mbp) was a mate-pair library with 3 kbp inserts and had an average read length of 389 bp. All sequencing was performed at the SciLifeLab SNP/SEQ sequencing facility at Uppsala University. Contigs from both runs were assembled with Newbler using a minimal overlap of 40 bp and 90% identity.

Phylogenetic binning of the contigs ≥1 kb was performed with PhylopythiaS^1^ (Patil et al., 2012) using the sample-specific model type. Additionally, contigs containing rRNA genes were identified by RNAmmer 1.2^2^ (Lagesen et al., 2007). The detected rRNA genes were phylogenetically assigned using the RDP Classifier (Wang et al., 2007). All contigs assigned to the *Epsilonproteobacteria* were reordered with the Mauve Aligner (Rissman et al., 2009) using the genome of *Sulfurovum* sp. NBC37-1 (Nakagawa et al., 2007) as scaffold (acc. no. NC_009663).

### Genome Annotation and Pathway Analysis

Reordered contigs were uploaded to the Micro Scope platform (v. 2.5.4, May 2014; Vallenet et al., 2013) and automatically annotated. Automatic annotation was manually edited using the microbial annotation system Magnifying Genome (MaGe; Vallenet et al., 2006) that includes PsortB, SwissProt, TrEMBL, and COGnitor. Metabolic pathways were predicted using the integrated pathway tools of MaGe that are based on the KEGG and MicroCyc databases. Genome completeness was estimated based on the MaGe Minimal Gene Set comprising 205 essential genes (Gil et al., 2004) and using the set of 139 conserved single copy genes (CSCGs) which occur only once in at least 90% of all bacterial genomes (Rinke et al., 2013).

The annotated contigs have been submitted to the European Nucleotide Archive (ENA) under the study accession no. PRJEB11632^3^.

## Results

### Genome Overview and Phylogenetic Assignment

Overall, the reconstructed population genome has a sequence length of around 1.6 Mb with a GC content of about 33%. Within 105 contigs, 1832 genomic objects with an average sequence length of about 850 bp were identified, comprising 30 tRNA genes, two not further specified RNA genes, and 1797 protein coding sequences (CDS). A 16S rRNA gene was detected showing 94% similarity to that of *Sulfurovum* sp. NCBI-37 and a 23S rRNA gene with 93% similarity to the same next relative. Additionally, a 5S rRNA gene was detected on the contig harboring the 23S rRNA gene. 1384 of the CDS belonged to at least one cluster of orthologous groups (COGs). **Table 1** summarizes the COG assignment. The genome completeness based on the Minimal Gene Set is 93% as 15 of the 205 genes are missing. Based on the CSCG set, the completeness is 97% (four genes of the 139 CSCG are missing). An overview of general genome characteristics is given in **Table 2**. The phylogenetic assignment of the population genome was analyzed based on Maximum Likelihood trees calculated from 16S rRNA gene sequences (only from cultured species) and DNA gyrase subunit A (encoded by *gyrA*) sequences of representative members of the class *Epsilonproteobacteria* (Supplementary Figure S1). Both trees show that the Zeitz epsilonproteobacterium forms a clade with members of the genus *Sulfurovum*. Based on the *gyrA* phylogeny, the next relative is *Sulfurovum* sp. AS07-7 which represents a *Sulfurovum*-like population genome retrieved from Acquasanta Terme (Hamilton et al., 2015).

**Table 1:** Number of coding DNA sequences (CDS) assigned to cluster of orthologous groups (COGs).

Process	Class ID	Description	CDS
Cellular processes and signaling	D	Cell cycle control, cell division, chromosome partitioning	28
	M	Cell wall/membrane/envelope biogenesis	118
	N	Cell motility	23
	O	Post-translational modification, protein turnover, chaperones	88
	T	Signal transduction mechanisms	51
	U	Intracellular trafficking, secretion, and vesicular transport	52
	V	Defense mechanisms	18
Information storage and processing	J	Translation, ribosomal structure, and biogenesis	144
	K	Transcription	68
	L	Replication, recombination, and repair	91
Metabolism	C	Energy production and conversion	129
	E	Amino acid transport and metabolism	131
	F	Nucleotide transport and metabolism	52
	G	Carbohydrate transport and metabolism	54
	H	Coenzyme transport and metabolism	82
	I	Lipid transport and metabolism	37
	P	Inorganic ion transport and metabolism	88
	Q	Secondary metabolites biosynthesis, transport, and catabolism	21
Poorly characterized	R	General function prediction only	221
	S	Function unknown	121

**Table 2:** General features of the reconstructed population genome.

Feature	
Genome length	1,625,596 bp
GC content	33%
Genome completeness	93–97%
No. of contigs	105
Average CDS length	850 bp
Protein coding density	89%
Genomic objects	1832
No. of CDS	1797
tRNA genes	30
rRNA genes	3
Other RNA genes	2

### Carbon Metabolism

**FIGURE 1:**
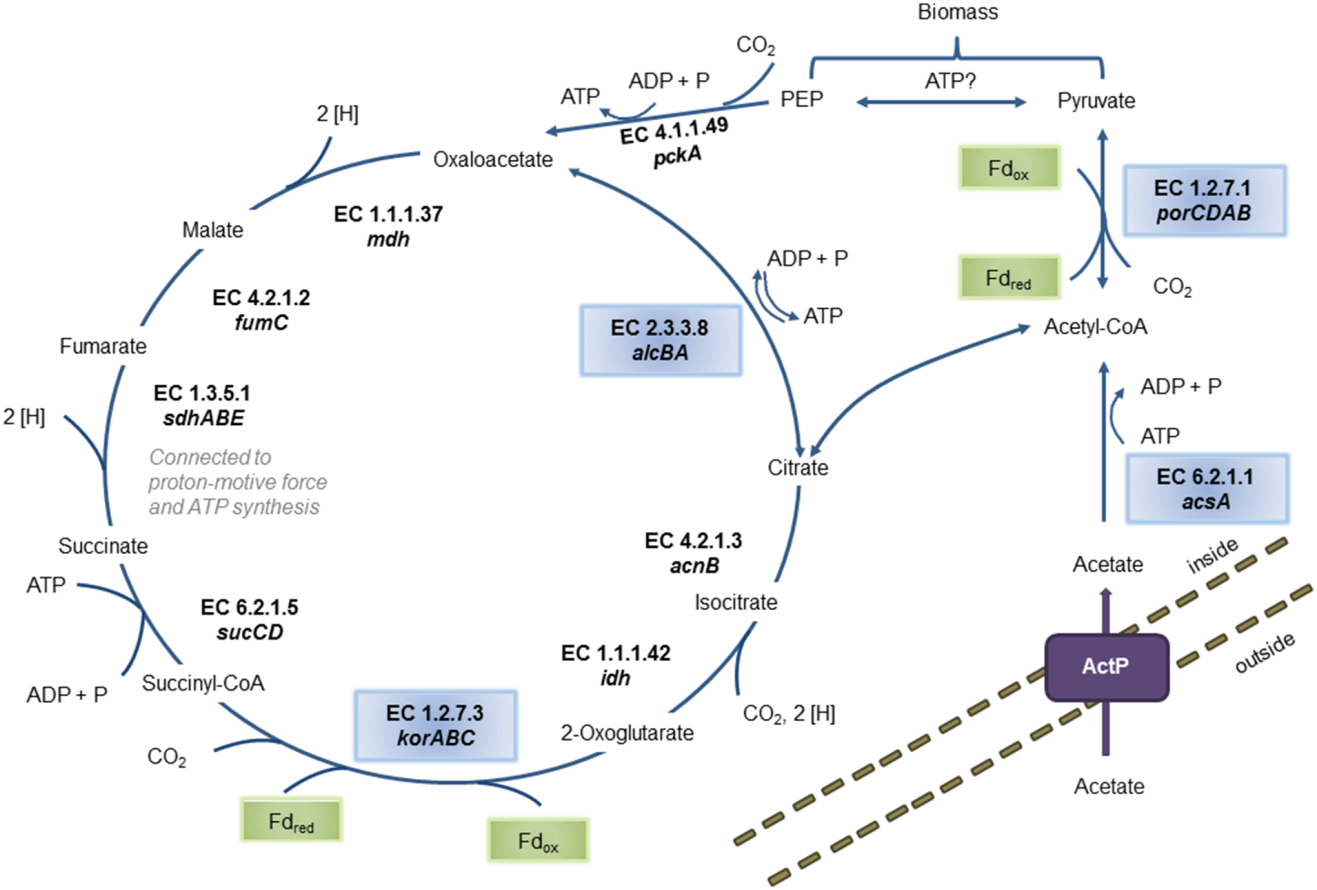
**Predicted pathways of biomass buildup from CO_2_ or from acetate.** The reconstructed population genome encodes two different mechanisms of carbon assimilation: either by importing acetate from the surrounding environment and activation via the acetyl-CoA synthetase AcsA (EC 6.2.1.1) or by CO_2_ fixation via the reductive citric acid (rTCA) cycle. Involved enzymes are: ATP-citrate lyase (EC 2.3.3.8), malate dehydrogenase (EC 1.1.1.37), fumarate hydratase (EC 4.2.1.2), fumarate reductase (EC 1.3.5.1), succinyl-CoA synthetase (EC 6.2.1.5), 2-oxoglutarate ferredoxin oxidoreductase (EC 1.2.7.3), NADP-dependent isocitrate dehydrogenase (EC 1.1.1.42) and citrate lyase (EC 4.2.1.3). The corresponding genes are denoted below the EC numbers. The two oxygen-sensitive ferredoxin-dependent CO_2_-fixation reactions are labeled in green boxes. Unique enzymes either part of the rTCA or involved in acetate processing are labeled in blue boxes. Activated acetyl-CoA is further processed to pyruvate and phosphoenolpyruvate (PEP) for the regeneration of rTCA intermediates or for biomass buildup. The enzymes pyruvate ferredoxin oxidoreductase (EC 1.2.7.1) and phosphoenolpyruvate carboxykinase (EC 4.1.1.49) are involved in this step. No enzyme catalyzing the reaction of pyruvate to PEP was detected in the population genome.

The genome contains all genes necessary for a complete rTCA cycle, indicating that the Zeitz epsilonproteobacterium can fix CO_2_ to build up biomass (**Figure 1**). All genes encoding the three key enzymes of the rTCA cycle were detected: *alcBA* encoding the ATP citrate lyase (EC 2.3.3.8), *korABC* encoding the 2-oxoglutarate ferredoxin oxidoreductase (EC 1.2.7.3), and *porCDAB* encoding the pyruvate ferredoxin oxidoreductase (EC 1.2.7.1). In this process, two CO_2_ molecules are fixed to synthesize one molecule of acetyl-CoA. A second source for acetyl-CoA is potentially the direct uptake of acetate and further processing to acetyl-CoA. The genes encoding the appropriate and unique enzyme acetyl-CoA synthetase AcsA (EC 6.2.1.1) and the acetate permease ActP were found in the genome. In a subsequent reaction, acetyl-CoA is converted to pyruvate by addition of a further CO_2_ molecule via the third key enzyme of the rTCA cycle, pyruvate ferredoxin oxidoreductase. A gene for the next step catalyzing the conversion of pyruvate to phosphoenolpyruvate (PEP) was not found. The gene *pckA* encoding a phosphoenolpyruvate carboxykinase (EC 4.1.1.49) was detected. PEP or pyruvate either regenerate the intermediates of the rTCA cycle or can be used for gluconeogenesis. Enzymes involved in the rTCA cycle participate also in other cell processes, such as the fumarate reductase/succinate dehydrogenase (EC 1.3.5.1) which can also function in fumarate respiration. In the genome, two fumarate reductases/succinate dehydrogenases are encoded on two different contigs (see next section).

### Energy Metabolism

**FIGURE 2:**
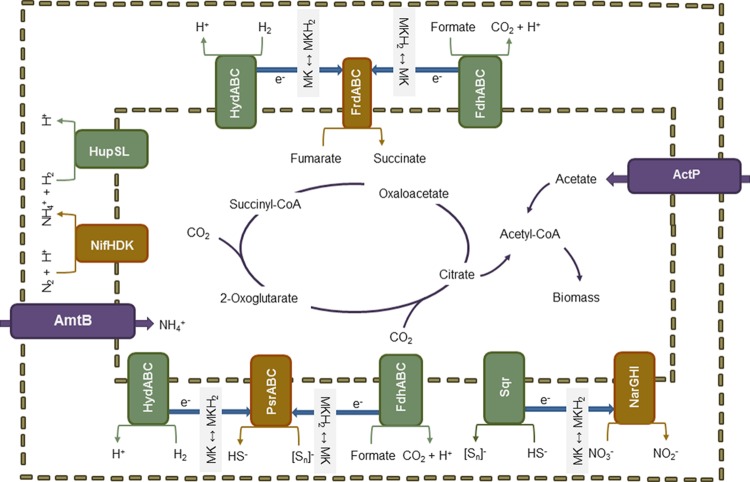
**Schematic overview about the predicted metabolic pathways.** The capability to fix nitrogen as alternative to ammonium uptake and to fix CO_2_ as alternative to acetate assimilation as well as the versatility regarding electron donors and acceptors might provide a selective advantage of the Zeitz epsilonproteobacterium in groundwater systems. Electron donors and acceptors are shown as redox couples as described in the literature. It is supposed that specifically the high affinity to acetate generated as intermediate of hydrocarbon degradation and a higher tolerance to sulfide accumulating due to sulfate reduction by *Deltaproteobacteria* define its ecological niche in the Zeitz aquifer.

An overview on the predicted pathways involved in energy metabolism and the corresponding electron donors and acceptors is shown in **Figure 2**. Genome analysis showed that hydrogen may function as electron donor. Two Ni-Fe containing hydrogenases are encoded in the genome, namely the uptake hydrogenase HupSL (EC 1.12.99.6) and the quinone-reactive hydrogenase HydABC (EC 1.12.5.1), both associated with the periplasmic membrane (Yates, 1988; Gross et al., 1998). The former is expressed under nitrogen-fixing conditions when hydrogen is generated and converted to minimize energy loss during fixation catalysis. Electrons released in this process are transferred to the ubiquinone pool. The oxidation of hydrogen via HydABC is coupled to the reduction of NAD^+^ and ferredoxin, establishing a proton gradient for ATP generation (Tsygankov, 2007). In addition, the gene *sqr* encoding a sulfide quinone oxidoreductase (EC 1.8.5.4) was found. This membrane-bound enzyme catalyzes the initial step in dissimilatory sulfide oxidation, the conversion of hydrogen sulfide to polysulfides (Schütz et al., 1999; Griesbeck et al., 2002). Genes for the complete oxidation of reduced sulfur species to sulfate via the Sox system were not detected. Furthermore, formate could serve to provide electrons. The gene *fdhA* encoding one of the three subunits of formate dehydrogenase (FDH) was identified (Bokranz et al., 1991). FDH oxidizes formate to carbon dioxide coupled with the reduction of NAD^+^ to NADH.

The Zeitz epsilonproteobacterium is a facultative anaerobe but has a strictly respiratory type of energy metabolism. The genome contains all genes necessary for oxidative phosphorylation with oxygen, including genes for an NADH-quinone oxidoreductase (EC 1.6.5.11), a succinate dehydrogenase/fumarate reductase (EC 1.3.5.1), a ubiquinol-cytochrome c reductase (EC 1.10.2.2), a cytochrome c oxidase cbb3-type (EC 1.9.3.1), an F-type ATPase (EC 3.6.3.14), and both ATPase-supporting enzymes polyphosphate kinase (*ppk*; EC 2.7.4.1) and inorganic pyrophosphatase (*ppa*; EC 3.6.1.1). In organisms exposed to oxygen, a mechanism to cope with oxygen-generated radicals would be expected. The *sodB* gene encoding a superoxide dismutase subunit (EC 1.15.1.1) was annotated, but the gene for the second subunit *sodA* is missing. A second oxygen protection mechanism is based on alkyl hydroperoxide reductase (EC 1.11.1.15). The respective gene *ahpC* was annotated. Under anoxic conditions, nitrate, polysulfide, or fumarate can serve as terminal electron acceptor for oxidative phosphorylation. For the reduction of nitrate to nitrite, *narG* and *narH* encoding a membrane-bound nitrate reductase were found (Bertero et al., 2003). The gene *narI* is putatively encoded in the CDS downstream of *narH*. No gene *narK* for the nitrate/nitrite transporter was found. Three genes are necessary for polysulfide reduction: *psrA* encoding the catalytic subunit PsrA, *psrB* encoding the electron-transferring subunit PsrB, and *psrC* encoding the membrane anchor PsrC (Jormakka et al., 2008). Complete *psrB* and *psrC* were found, but only a fragment of *psrA* was identified. In *Epsilonproteobacteria*, the fumarate reductase involved in fumarate respiration consists of three subunits FrdABC. FrdA is the catalytic subunit, FrdB contains iron-sulfur clusters, and FrdC the cytochrome transferring the electrons to carriers and anchoring the enzyme in the membrane (Kröger et al., 2002). As mentioned above, two copies of succinate dehydrogenase/fumarate reductase are encoded in the genome of the Zeitz epsilonproteobacterium. One copy comprises only the genes for FrdA and FrdB, whereas the other copy contains the genes for all three subunits.

### Sulfur and Nitrogen Assimilation

No sulfate uptake system was detected, except for *yvdB* encoding a subunit of a sulfate transporter-like protein. Furthermore, the genome harbors the *sat* gene for sulfate adenylyltransferase (EC 2.7.7.4) which is responsible for the activation of sulfate to adenylyl sulfate (APS). Alternatively, sulfide rather than sulfate could be assimilated. Both enzymes necessary for sulfur assimilation from sulfide, serine *O*-acetyltransferase (EC 2.3.1.30) and cysteine synthase A (EC 2.5.1.47), are encoded in the population genome.

Genes encoding nitrogen fixation and ammonium uptake were identified. A dinitrogenase responsible for nitrogen fixation is encoded by *nifHDK* (EC 1.18.6.1). The MoFe-protein NifDK is the site for nitrogen reduction and the Fe-protein NifH transfers electrons (Raymond et al., 2004). As a result of this catalytic process, ammonia is synthesized and fed into metabolic pathways. For an active dinitrogenase complex, further *nif* genes are required. Distributed over the genome, eighteen genes were assigned as related to nitrogen fixation (**Table 3**). The *nifEN* gene products act as scaffolding agents for cofactors, NifWZ is related to catalytic stability, and NifVB, NifQ as well as NifT are involved in the biosynthesis of dinitrogenase subunits (Böhme, 1998). Regulators of gene expression are encoded by *nifA, nifX, nifU* (Dixon et al., 1980; Gosink et al., 1990; Fu et al., 1994). Furthermore, *amtB* encoding an ammonia importer was detected. Ammonia, imported or produced via nitrogen fixation, is subsequently funneled into the amino acid metabolism via glutamine synthetase (EC 6.3.1.2) and glutamate synthase (EC 1.4.1.13), or glutamate dehydrogenase (EC 1.4.1.4). All of the respective genes are present in the genome.

**Table 3:** Genes involved in nitrogen assimilation.

Gene	Function	Reference
*nifT*	Maturation	Simon et al., 1996
*nifH*	Fe dinitrogenase reductase	Steenhoudt and Vanderleyden, 2000
*nifD*	FeMo dinitrogenase	Steenhoudt and Vanderleyden, 2000
*nifK*	FeMo dinitrogenase	Steenhoudt and Vanderleyden, 2000
*nifE*	Scaffold for FeMo-cofactor	Raymond et al., 2004
*nifN*	Scaffold for FeMo-cofactor	Raymond et al., 2004
*nifB*	Biosynthesis of FeMo-cofactor	Temme et al., 2012
*glnB*	N-signal transmitter protein PII	Steenhoudt and Vanderleyden, 2000
*nifU*	Biosynthesis FeMo-cofactor	Böhme, 1998
*nifX*	Negative regulator	Gosink et al., 1990
*nifA*	Transcriptional activator	Steenhoudt and Vanderleyden, 2000
*nifW*	Catalytic stability	Böhme, 1998
*nifZ*	Catalytic stability	Böhme, 1998
*nifQ*	Biosynthesis of FeMo-cofactor	Temme et al., 2012
*nifV*	FeMo-cofactor	Böhme, 1998

### Transport Systems

Over 30 genes were assigned as related to ABC transporters. The specificity in annotation of these genes varied making it difficult to assign a (clear) function to all encoded transporters specifically. Those with definite assignment comprise the molybdenum transporter encoded by *modABCDE*, the energy-consuming TonB transport system consisting of *tonB, exbB* and *exbD*, the specific phosphate transporter Pst encoded by *pstSCAB* and the corresponding regulators *phoU* and *phoB.* Furthermore, uptake complexes for potassium (*ktrAB*), ferrous iron (*feoAB* with the corresponding regulator gene *fur)*, and zinc (*znuBCA*) were detected. The export of large organic molecules from the cell may be accomplished using the type II secretion system together with the translocation pathways Tat and Sec-SRP. Almost all genes for both translocation pathways are present in the genome. The *secM* gene of the Sec SRP pathway and some subunits of the membrane crossing type II transporter are missing. Additionally, *ffh* encoding a signal recognition particle and targeting proteins for relocation was identified.

## Discussion

### Genome Size and Completeness

The typical genome sizes of *Epsilonproteobacteria* from comparable habitats range from 2.1 to 3.0 Mb (Baar et al., 2003; Miller et al., 2007; Sievert et al., 2008; Grote et al., 2012; Handley et al., 2014). The G+C content of epsilonproteobacterial genomes varies between 32 and 48% (Baar et al., 2003; Miroshnichenko et al., 2004). With 33% G+C content and a size of 1.6 Mb, the Zeitz epsilonproteobacterial population genome seems to be within the lower range within this group. However, the reconstructed population genome is incomplete as shown by the completeness estimation based on the Minimal Gene Set and the CSCG set. Moreover, only 30 tRNA genes were detected compared to up to 47 tRNA genes in *Sulfurimonas gotlandica* str. GD1 (Grote et al., 2012) and just one copy of 16S rRNA and 23S+5S rRNA genes was identified, whereas typically around three copies of rRNA operons are found in epsilonproteobacterial genomes (Sievert et al., 2008). Consequently, the present annotation likely does not cover the complete metabolic capabilities, resulting potentially in pathway gaps and missing essential metabolic functions. Thus, the annotation results need to be interpreted with caution, at least regarding the absence of metabolic functions. Nevertheless, the present annotated genes provide important insights into the metabolic versatility of the Zeitz epsilonproteobacterium as discussed below.

### Metabolic Versatility in Carbon Source and Energy Supply

*Epsilonproteobacteria* are known to possess the capability of carbon fixation establishing them as important primary producers in oligotrophic environments and underscoring their ability to adapt to carbon-limited habitats (Hügler et al., 2005; Grote et al., 2012; Stokke et al., 2015). Overall, six pathways for autotrophic carbon fixation have been described: the Calvin-Benson reductive pentose phosphate cycle, the rTCA cycle, the reductive acetyl coenzyme A (Wood-Ljungdahl) pathway, the 3-hydroxypropionate bi-cycle, the 3-hydroxypropionate/4-hydroxybutyrate cycle, and the dicarboxylate/4-hydroxybutyrate cycle (Berg, 2011). The rTCA cycle has been described only for a few bacterial groups such as green sulfur bacteria and *Deltaproteobacteria*, but it seems to be widely distributed among *Epsilonproteobacteria* (Hügler et al., 2005; Campbell et al., 2006; Sievert et al., 2008; Handley et al., 2014). Correspondingly, the Zeitz epsilonproteobacterium genome encodes a complete rTCA cycle, including the genes for the key enzymes ATP citrate lyase, pyruvate ferredoxin oxidoreductase and 2-oxoglutarate ferredoxin oxidoreductase (Evans et al., 1966). The fumarate reductase/succinate dehydrogenase is also part of the rTCA cycle and two copies of this enzyme are present in the genome with three and two subunits, respectively. A similar observation was reported by Sievert et al. (2008). They suggested the two-subunit fumarate reductase/succinate dehydrogenase to be cytoplasmic and involved in the rTCA cycle.

As an alternative to an inorganic carbon source, the potential for direct acetate assimilation is given which requires the expression of *actP* acting as acetate import system and the acetyl-CoA synthetase gene *acsA* responsible for acetate activation (Berg, 1956; Gimenez et al., 2003). Considering the presence of a complete rTCA cycle and the alternative of direct acetate import and assimilation, the organism is equipped for a mixotrophic lifestyle, which was proposed for other *Epsilonproteobacteria* as well (Campbell et al., 2006). Recently, a time-resolved protein-SIP experiment with ^13^C_2_ acetate, daily spiked at a concentration of 10 μM to the benzene-degrading consortium, revealed that the Zeitz epsilonproteobacterium exhibited the fastest and highest incorporation of labeled carbon from acetate compared to other acetate utilizers, indicating its role as a primary and highly efficient acetate scavenger within the syntrophic consortium (Starke et al., in revision). Mixotrophy which can be inferred from the genome and the capability of highly efficient acetate capture as demonstrated by Starke et al. (in revision) are metabolic traits which can confer selective advantages in groundwater habitats and in particular in hydrocarbon-contaminated aquifers, where acetate is formed as metabolite of syntrophic hydrocarbon degradation or is leaked out in small concentrations from the anaerobic degradation of organic compounds (Rakoczy et al., 2011).

Different combinations of organic and inorganic electron donors and acceptors have been described for *Epsilonproteobacteria* (Campbell et al., 2006; Kern and Simon, 2009). An overview of the potential electron donors and acceptors according to the genome annotation is given in **Table 4**. Since the Zeitz epsilonproteobacterium was enriched from a sulfidic environment and under sulfate-reducing conditions, the most obvious electron donor would be sulfide. The oxidation of reduced S-species to sulfate via the Sox system has frequently been observed in marine *Epsilonproteobacteria* (Nakagawa et al., 2007; Sievert et al., 2008; Grote et al., 2012). However, genes for the Sox system were not found in the present (incomplete) genome. Sulfide oxidation to polysulfide using sulfide quinone oxidoreductase (SQR) was suggested for the betaproteobacterium *Thiobacillus denitrificans* which additionally contains also parts of the Sox pathway and may oxidize sulfide simultaneously by SQR and Sox under nitrate-reducing conditions (Beller et al., 2006a,b). This oxidation could be coupled via menaquinone with the polysulfide reductase PsrABC which has the capability to reduce polysulfides. The respective *psr* operon was found in the genomes of other *Epsilonproteobacteria* as well, and Psr activity was detected (Yamamoto et al., 2010; Grote et al., 2012). Such coupling generates only limited energy, but might serve for other purposes like an internal sulfur cycle. Alternatively, polysulfide reduction may be linked to hydrogen or formate oxidation with menaquinone again acting as electron carrier (Hedderich et al., 1999; Takai et al., 2005; Yamamoto et al., 2010). However, only the subunit gene *fdhA* of the FDH was explicitly annotated whereas all genes for the respective hydrogenase (*hydABC*) were found. Hydrogen and formate oxidation might be also coupled to fumarate reduction, a respiration type which is well-investigated for *Epsilonproteobacteria* such as *Wolinella* (Kröger et al., 1992). Electrons released via hydrogen/formate oxidation to build up the proton gradient are transferred via menaquinone to fumarate reductase. Fumarate reduction in terms of energy metabolism is a membrane-bound process (Kröger et al., 1992). Hence, the second copy of a fumarate reductase/succinate dehydrogenase annotated in the genome with three subunits could be the appropriate enzyme for this reaction. Sources for fumarate could be degraded carbohydrates or proteins whereby it is unclear if the degradation is an inner cell process or fumarate is taken up from the surrounding medium (Kröger et al., 1992). Specific dicarboxylate transporters (DcuAB) for fumarate uptake (Ullmann et al., 2000) were not identified in the present genome, but non-specifically annotated transporter genes could encode an appropriate uptake system (Baar et al., 2003). Theoretically, if rTCA cycle and acetate assimilation work in parallel, fumarate could be internally generated by diverting PEP into the rTCA cycle (see **Figure 1**). However, the respiration of internally produced fumarate with hydrogen/formate or sulfide as electron donor would probably not generate net ATP allowing growth. The conversion of acetate to fumarate costs at least 1 mol ATP per mol acetate (**Figure 1**), whereas fumarate reduction with hydrogen/formate or sulfide probably produces less than 1 mol ATP per fumarate (Kröger et al., 2002).

**Table 4:** Potential electron acceptors and donors and their redox potentials.

Electron acceptor	ΔE (mV)	Reference
NO_3_^-^ → NO_2_^-^	+433	Thauer et al., 1977
[S_n_]^2-^ → HS^-^	+260	Dietrich and Klimmek, 2002
Fumarate → Succinate	+30	Kröger et al., 1992

**Electron donor**	**Δ E (mV)**	**Reference**

H_2_ → H^+^	-414	Dietrich and Klimmek, 2002
Formate → CO_2_	-432	Thauer et al., 1977
HS^-^ → [S]	-275	Dietrich and Klimmek, 2002

Similar to enzymes of the rTCA cycle, the hydrogenase HydABC is a ferredoxin-dependent enzyme coupling hydrogen oxidation with the reduction of NAD^+^ and ferredoxin (Wang et al., 2013). Thus, this enzyme is oxygen-sensitive showing that the Zeitz epsilonproteobacterium is adapted to anoxic or microoxic conditions. However, the genetic capability to utilize low levels of oxygen exists as all genes necessary for aerobic respiration are present in the genome. This circumstance was also described for *Candidatus* Sulfuricurvum sp. in a previous study (Handley et al., 2014). Another study proved growth of *Sulfuricurvum kujiense* under microaerobic conditions (Kodama and Watanabe, 2004). Similar results were obtained with *Sulfurovum* sp. NBC37-1 which is the next cultured relative of the Zeitz epsilonproteobacterium and was found to grow with various combinations of electron donors and acceptors, e.g., hydrogen and oxygen (Yamamoto et al., 2010).

Another common electron acceptor for *Epsilonproteobacteria* is nitrate (Miroshnichenko et al., 2004; Brettar et al., 2006; Nakagawa et al., 2007). The genome studied here encodes the nitrate reductase NarGHI. Growth tests in the studies of Yamamoto et al. (2010) and Handley et al. (2014) showed positive results for various combinations including coupling with hydrogen or sulfide as electron donor. Although no nitrate importer gene was detected in the present genome, nitrate reduction could be a viable option since the redox potential of nitrate to nitrite is relatively positive compared to other anaerobic terminal electron acceptor processes (see **Table 4**). Regarding the ecological niche in contaminated groundwater, nitrate can be relevant and *Epsilonproteobacteria* are known to be involved in nitrate-dependent reoxidation of reduced sulfur compounds (Yamamoto and Takai, 2011). However, the capability of nitrate respiration does not explain the ecophysiology of the Zeitz epsilonproteobacterium in our enrichment cultures as the growth media did not contain any nitrate.

### Pathways for Sulfur and Nitrogen Assimilation

Besides its role as an electron donor, sulfide plays a role as a nutrient in bacterial cells. Based on the metagenome data, the Zeitz epsilonproteobacterium cannot assimilate sulfate; genes for an appropriate sulfate import system are missing as well as genes involved in the sulfate activation pathway except of the *sat* gene which encodes ATP sulfurylase catalyzing the formation of adenosine 5′-phosphosulfate (Kreddich, 1996). Although it cannot be rules out that the enzymes for assimilatory sulfate reduction are encoded in the missing parts of the genome, the direct assimilation of sulfide by the cysteine synthase saves energy and is thus plausible in sulfidic environments. Notably, other *Epsilonproteobacteria* have been described to be capable of assimilating sulfate (Baar et al., 2003). It is conspicuous that the Zeitz epsilonproteobacterium harbors a *sat* gene but possibly no other genes for assimilatory sulfate reduction. One could speculate that this capability was lost during adaptation to sulfidic environments with a shift toward a specialization in sulfide as energy and sulfur source.

For nitrogen assimilation, ammonium can be directly used due to the presence of *amtB*. AmtB is a membrane uptake protein importing ammonium into the cell at low concentrations (Khademi et al., 2004; Zheng et al., 2004). In analogy to the use of sulfide as sulfur source, the direct use of ammonia as nitrogen source might be an adaptation to an anaerobic lifestyle as the energy-consuming reduction of oxidized N species is avoided. Notably, dinitrogen fixation is not common in *Epsilonproteobacteria* and was so far only described for *Wolinella succinogenes* (Baar et al., 2003) and *Arcobacter nitrofigilis* (McClung et al., 1983). Nitrogen fixation provides a selective advantage in nitrogen-limited habitats even though this process is very energy-demanding. The nitrogen fixation is linked to hydrogen formation. Hence, recovery of energy via hydrogen oxidation minimizes the loss of energy during the nitrogen fixation process. The subunits for the appropriate hydrogenase (HupSL) are encoded in the genome. Combining N-fixation with hydrogen oxidation to save energy has been described for *Cyanobacteria* (Tamagnini et al., 2000; Schütz et al., 2004) but also mentioned for other *Proteobacteria* such as *Allochromatium* (Weissgerber et al., 2011) and *Rhodospirillum* (Kern et al., 1994).

### Oxygen-sensitive Enzymes and Oxygen Tolerance

Enzymes specific for the rTCA cycle are dependent on the interaction with ferredoxin, an extremely oxygen-sensitive electron carrier (Bruschi and Guerlesquin, 1988). Both oxidoreductases (2-oxoglutarate ferredoxin oxidoreductase and pyruvate ferredoxin oxidoreductase) are ferredoxin-dependent enzymes and thus highly sensitive to oxygen (Evans et al., 1966). Other ferredoxin-dependent enzymes are directly inhibited in the presence of oxygen such as the nitrogen-fixing nitrogenase (Fay, 1992). Simultaneously, the genome encodes the complete oxidative phosphorylation pathway to generate energy via oxygen reduction. Taking both circumstances into account, the Zeitz epsilonproteobacterium could potentially tolerate oxygen and grow under microoxic conditions as described for other representatives of this class (Miller et al., 2007; Sievert et al., 2008; Bronowski et al., 2014; Handley et al., 2014). To handle excess oxygen which may form radicals within the cell and interfere with fundamental pathways such as carbon fixation, two mechanisms for radical scavenging are likely. Indeed, only *sodB* encoding the superoxide dismutase subunit converting superoxide radicals into hydrogen peroxide and water (Störz et al., 1990) was found in the genome. The *sodA* gene is missing as well as the gene for the subsequent catalase, a circumstance which was previously described for *Epsilonproteobacteria* (Bronowski et al., 2014). This limitation might be overcome by the alkyl hydroperoxide reductase AhpC, which alternatively scavenges hydrogen peroxides (Seaver and Imlay, 2001; Cosgrove et al., 2007).

### Transport Systems

Several uptake transporters and exporting pathways are encoded in the genome and are crucial for the organism. The molybdenum transporter ModABC is essential for nitrogen-fixing organisms since the nitrogenase depends on molybdenum (Chan et al., 1993; Allen et al., 1994). Furthermore, the specific phosphate transporter PstSCAB for environments with low phosphate concentrations was detected (Ames, 1986). For its induction, regulators are needed which both were found adjacent to the *pst* operon. Thereby, the function of PhoU is not completely understood, but it is likely involved in an interaction of the two-component signal system PhoRB (Steed and Wanner, 1993; Wanner et al., 1996) and transporter subunit PstB (Gardner et al., 2014). Other nutrient uptake systems such as a potassium transporter with the subunit genes *ktrB* and *krtC* (Holtmann et al., 2003) or the zinc transporter ZnuBCA (Patzer and Hantke, 1998) are also encoded. The genome analysis suggests that the Zeitz epsilonproteobacterium might have different strategies to gather iron from the environment. Ferric iron is insoluble under pH neutral conditions and Gram-negative bacteria use the energy-consuming TonB transporter to import ferric iron by binding the ion to chelating siderophores (Moeck and Coulton, 1998). The gene *tonB* encodes the protein to transduce energy derived from a proton motive force for the energy-consuming translocation, and *exbB* and *exbD* encode proteins responsible for restoring the conformational structure of TonB. Since the Zeitz epsilonproteobacterium originates from an anaerobic environment, the major form of iron is ferrous iron. Under these conditions, the encoded ferrous iron uptake complex FeoAB acts as supply system for iron (Kammler et al., 1993; Kim et al., 2012).

Besides import pathways, the export of proteins or toxic metabolites from the cytoplasm to the extracellular space is of similar importance. The function of protein secretion is likely carried out by the type II secretion system which is common among Gram-negative bacteria (Korotkov et al., 2012). The genes for several subunits of the type II secretion apparatus are missing but the presence of genes for Sec- and Tat-dependent translocation can be seen as a clear hint for its use. Both the Sec and Tat systems for the translocation across the inner membrane interact with the type II secretion pathway to transport proteins from the periplasm out of the cell (Voulhoux et al., 2001).

### Ecological Niche of the Zeitz Epsilonproteobacterium

The Zeitz epsilonproteobacterium shares some metabolic features with other members of this class, such as the capabilities to fix CO_2_ via the rTCA cycle (Takai et al., 2006), to utilize hydrogen as energy source (Takai et al., 2003, 2006), to use sulfur compounds as electron acceptors and donors (Takai et al., 2003; Yamamoto and Takai, 2011; Handley et al., 2014), and to tolerate oxygen on microoxic levels (Kodama and Watanabe, 2004; Nakagawa et al., 2007). A feature not described for other *Epsilonproteobacteria* is the genomic capability to gather carbon via both, acetate assimilation and carbon dioxide fixation, which means in consequence a potentially mixotrophic lifestyle. Acetate assimilation is a rare feature for *Epsilonproteobacteria* (Kodama et al., 2007; Sievert et al., 2008). Also the capability to fix dinitrogen is rarely described for representatives of this group (McClung et al., 1983; Baar et al., 2003). Nevertheless, there are still gaps in the annotated genome potentially hiding many genomic properties which cannot be considered so far.

The Zeitz epsilonproteobacterium originates from a sulfidic benzene-contaminated aquifer and was enriched as member of a syntrophic community. The key player, a *Pelotomaculum* sp. initially attacks benzene and shares carbon and electrons from benzene degradation with other community members belonging to several *Deltaproteobacteria* and the epsilonproteobacterium. The whole degradation is coupled to sulfate reduction as terminal electron acceptor process (Kleinsteuber et al., 2008; Herrmann et al., 2010). It was unclear why the Zeitz epsilonproteobacterium persisted in this enriched consortium or even increased in its abundance, considering that *Epsilonproteobacteria* are not known to use sulfate as electron acceptor, and the involved *Deltaproteobacteria* are potentially capable to consume and mineralize all intermediates from syntrophic benzene degradation such as acetate, hydrogen, or formate. In DNA-SIP experiments with ^13^C_6_-labeled benzene, the epsilonproteobacterium was shown to incorporate ^13^C over time. It was supposed that acetate as a putative intermediate of syntrophic benzene degradation may serve as carbon source for the epsilonproteobacterium (Herrmann et al., 2010). This hypothesis was not supported by the respective protein-SIP experiment with ^13^C_6_-labeled benzene (Taubert et al., 2012), probably due to the low abundance of *Epsilonproteobacteria* in those cultures and the low coverage of epsilonproteobacterial genes in the metagenome dataset applied for peptide identification. However, the recently performed protein-SIP experiment spiking fully ^13^C_2_-labeled acetate in addition to the ongoing mineralization of unlabeled benzene (Starke et al., in revision) as well as the presence of genes for an acetate importer and for acetate activation confirm the hypothesis that the Zeitz epsilonproteobacterium is a key acetate scavenger within the consortium.

The capability to use various electron donors such as hydrogen, formate and sulfide, and various electron acceptors such as oxygen, nitrate, polysulfide, and fumarate provides the metabolic versatility to colonize different groundwater environments. Although nitrate and oxygen may be present in upper groundwater levels at the fringe of the contamination plume of the Zeitz aquifer, they were neither relevant in central and lower parts of the plume where the samples for enrichment cultures were taken from, nor present in the sulfate-reducing enrichment cultures. The Zeitz site has been contaminated with hydrocarbons since 70 years or longer (Schirmer et al., 2006). Hence, strictly anoxic, sulfidic conditions may have persisted for decades. Thus, fumarate and polysulfide are assumed to be relevant electron acceptors, whereas hydrogen, formate, and sulfide are the likely electron donors. Except for the redox couple polysulfide – sulfide, each combination is theoretically feasible (see **Table 4**). Hydrogen or formate is formed during syntrophic benzene degradation and sulfide is generated during growth of the sulfate-reducing *Deltaproteobacteria* (Kleinsteuber et al., 2008). It can be assumed that the hydrogenotrophic *Deltaproteobacteria* outcompete the epsilonproteobacterium for hydrogen as electron donor due to a proposed higher affinity to hydrogen (Cord-Ruwisch et al., 1988; Rakoczy et al., 2011). However, at higher sulfide levels which are toxic to other bacteria, the epsilonproteobacterium might get a selective advantage due to a higher sulfide tolerance, rendering hydrogen oxidation feasible. For instance, benzene degradation by a sulfate-reducing consortium was inhibited at sulfide concentrations above 1.5 mM (Taubert et al., 2012) whereas *Epsilonproteobacteria* oxidizing sulfide with nitrate as electron acceptor have been described to tolerate sulfide levels of 2 mM (Poser et al., 2014) or even 3 mM (Gevertz et al., 2000). This is in accordance with the observation that the Zeitz epsilonproteobacterium increases in abundance in aromatics-degrading batch cultures which contain high sulfide levels (Bozinovski et al., 2012). Besides hydrogen or formate, sulfide is a common electron donor for *Epsilonproteobacteria* (Campbell et al., 2006). The generation of polysulfide might be a result of sulfide oxidation, potentially leading to the formation of elemental sulfur which reacts spontaneously to polysulfide (Streudel, 1996). Elemental sulfur/polysulfide could serve in reverse as electron acceptor. Alternatively, electrons are transferred to fumarate as part of the rTCA cycle. Potential sources of fumarate could be an internal process of a combined rTCA cycle and acetate assimilation, or fumarate released by other bacteria in the community.

The capability to assimilate nitrogen by direct import of ammonium via AmtB is characteristic for the adaptation to groundwater systems in which ammonium is the major nitrogen source. Ammonium was also provided as nitrogen source in the enrichment cultures. The capability to fix dinitrogen might be a selective advantage in habitats generally depleted in nutrients, such as aquifers impacted by massive hydrocarbon input and biodegradation.

Summarizing the genomic features of the Zeitz epsilonproteobacterium, this organism is well-adapted to pristine and hydrocarbon-contaminated sulfidic aquifers considering the mixotrophic lifestyle, the type of energy metabolism, and the mechanisms of nitrogen and sulfur assimilation. It can potentially adapt to changing environmental conditions (microoxic, altered redox conditions or carbon and nitrogen sources). The most striking features defining its ecophysiological role in the hydrocarbon-degrading, sulfate reducing consortia are the high affinity to acetate and the high tolerance to sulfide which can simultaneously be used for assimilatory and dissimilatory purposes. Thus, the Zeitz epsilonproteobacterium may occupy a specific ecological niche in the intermediary ecosystem metabolism of hydrocarbon-contaminated sulfidic subsurface habitats by metabolizing the key intermediate acetate and recycling sulfide which cannot be consumed by other community members. The latter might be the reason for the co-occurrence or even mutualistic relationship with sulfate-reducing *Deltaproteobacteria*. However, the hypothesized role needs to be proven in cultivation experiments. The annotated population genome provides clues on the possible enrichment strategies for isolating the Zeitz epsilonproteobacterium which will be applied in future experiments.

## Author Contributions

AK performed the genome annotation, interpreted the data, and drafted the paper. KS designed the study, analyzed sequence data, contributed to annotation and interpretation of the genome data, and critically revised the paper. RS contributed to genome annotation and interpretation of the data, and contributed to writing the paper. SB performed the metagenome sequencing, analyzed sequence data, and contributed to writing the paper. CV designed the study, interpreted the data and wrote the paper. SK designed the study, contributed to sequence analysis, genome annotation and interpretation of the sequence data and wrote the paper.

## Conflict of Interest Statement

The authors declare that the research was conducted in the absence of any commercial or financial relationships that could be construed as a potential conflict of interest.

## Footnotes

1 http://binning.bioinf.mpi-inf.mpg.de/

2 http://www.cbs.dtu.dk/services/RNAmmer/

3 www.ebi.ac.uk/ena/data/view/PRJEB11632
