# Supplementary material for: Metagenome-Based Metabolic Reconstruction Reveals the Ecophysiological Function of Epsilonproteobacteria in a Hydrocarbon-Contaminated Sulfidic Aquifer
Source: Front Microbiol. 2015 Dec 10;6:1396. doi: 10.3389/fmicb.2015.01396 (PMC4674564; doi:10.3389/fmicb.2015.01396)
Supplement: Supplementary file 1 [file Image_1.PDF]

Supplementary Material

Metagenome-based metabolic reconstruction reveals the ecophysiological function of *Epsilonproteobacteria* in a hydrocarbon-contaminated sulfidic aquifer

Andreas H. Keller[1,2], Kathleen M. Schleinitz[2], Robert Starke[3], Stefan Bertilsson[4], Carsten Vogt[1], Sabine Kleinsteuber[2]*

[1] Department of Isotope Biogeochemistry, Helmholtz Centre for Environmental Research – UFZ, Leipzig, Germany

[2] Department of Environmental Microbiology, Helmholtz Centre for Environmental Research – UFZ, Leipzig, Germany

[3] Department of Proteomics, Helmholtz Centre for Environmental Research – UFZ, Leipzig, Germany

[4] Department of Ecology and Genetics, Limnology and Science for Life Laboratory, Uppsala University, Uppsala, Sweden

*Correspondence:
Dr. Sabine Kleinsteuber
Helmholtz Centre for Environmental Research – UFZ
Department of Environmental Microbiology
Permoserstr. 15
04318 Leipzig, Germany
sabine.kleinsteuber@ufz.de

Running title: *Epsilonproteobacteria* in a sulfidic aquifer

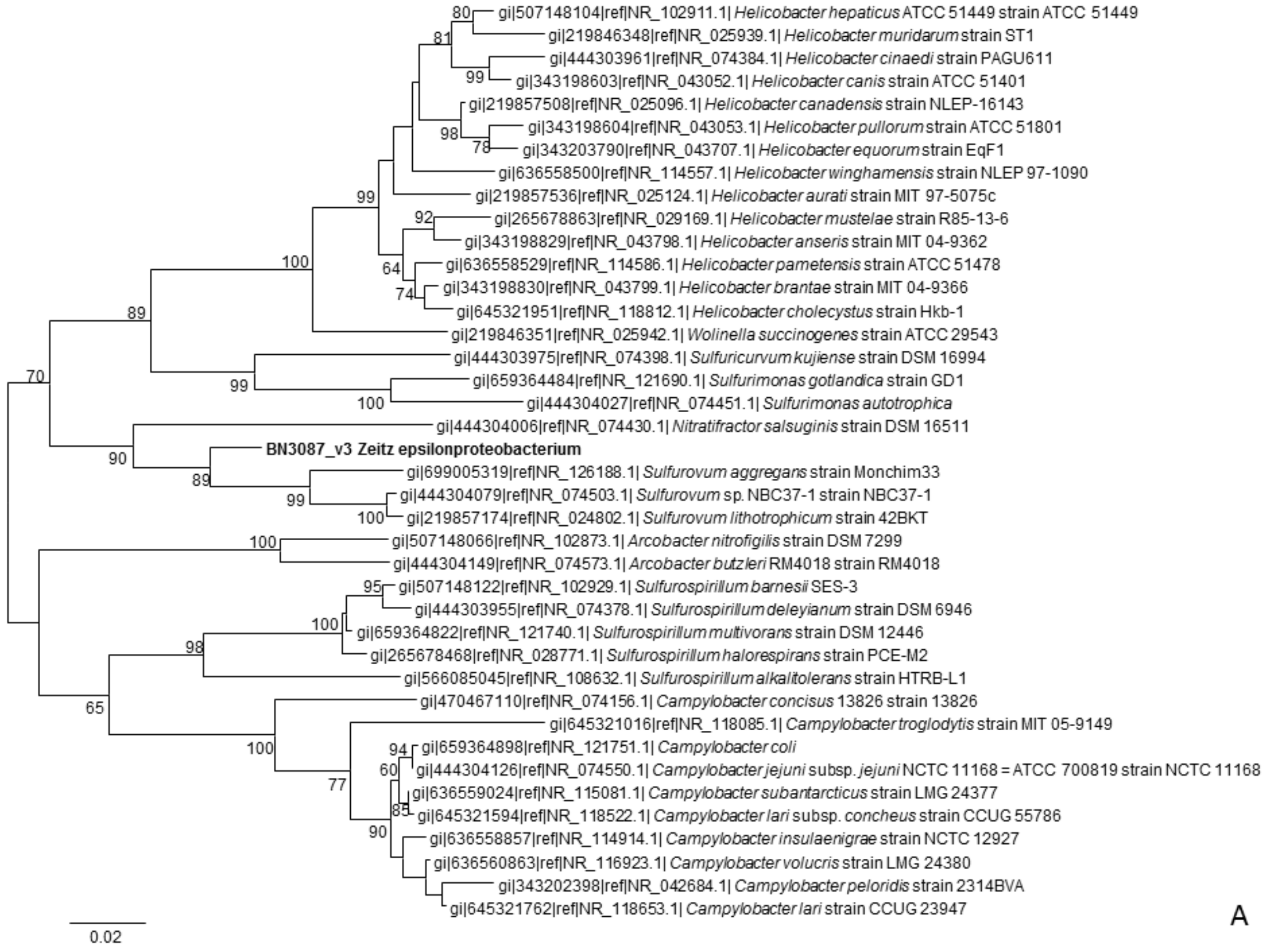

Supplementary Figure 1A. Maximum Likelihood phylogenetic tree of 16S rRNA gene sequences from the Zeitz epsilonproteobacterium (printed in bold) and representative species affiliated to the *Epsilonproteobacteria*. Nucleotide sequences were aligned with ClustalW (Thompson et al., 1994). The evolutionary history was inferred by using the Maximum Likelihood method based on the Tamura-Nei model (Tamura and Nei, 1993). The tree with the highest log likelihood (-10702.2693) is shown. The percentage of trees in which the associated taxa clustered together is shown next to the branches (only percentages >50% are shown). Initial trees for the heuristic search were obtained automatically by applying Neighbor-Join and BioNJ algorithms to a matrix of pairwise distances estimated using the Maximum Composite Likelihood approach, and then selecting the topology with superior log likelihood value. The tree is drawn to scale, with branch lengths measured in the number of substitutions per site. The analysis involved 40 nucleotide sequences. All positions containing gaps and missing data were eliminated. There were a total of 1337 positions in the final dataset. Evolutionary analyses were conducted in MEGA6 (Tamura et al., 2013).

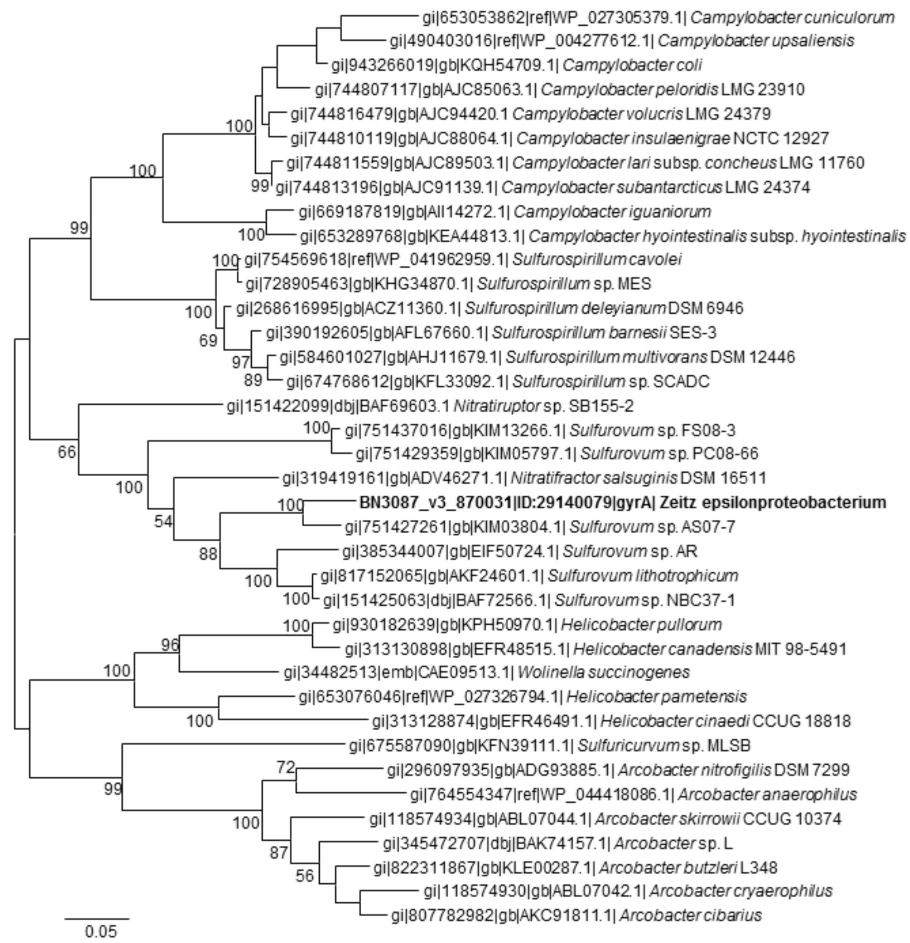

B

Supplementary Figure 2B. Maximum Likelihood phylogenetic tree of DNA gyrase subunit A (*gyrA*) amino acid sequences from the Zeitz epsilonproteobacterium (printed in bold) and representative species affiliated to the *Epsilonproteobacteria*. The evolutionary history was inferred by using the Maximum Likelihood method based on the Poisson correction model (Zuckerkandl and Pauling, 1965). The tree with the highest log likelihood (-17254.1570) is shown. The percentage of trees in which the associated taxa clustered together is shown next to the branches (only percentages >50% are shown). Initial trees for the heuristic search were obtained by applying the Neighbor-Joining method to a matrix of pairwise distances estimated using a JTT model. The tree is drawn to scale, with branch lengths measured in the number of substitutions per site. The analysis involved 38 amino acid sequences. All positions containing gaps and missing data were eliminated. There were a total of 798 positions in the final dataset. Evolutionary analyses were conducted in MEGA6 (Tamura et al., 2013).

References

Tamura, K. and Nei, M. (1993). Estimation of the number of nucleotide substitutions in the control region of mitochondrial DNA in humans and chimpanzees. *Mol. Biol. Evol.* 10, 512 - 526.

Tamura, K., Stecher, G., Peterson, D., Filipski, A., and Kumar, S. (2013). MEGA6: Molecular Evolutionary Genetics Analysis version 6.0. *Mol. Biol. Evol.* 30, 2725 - 2729.

Thompson, J.D., Higgins, D.G., and Gibson, T.J. (1994). CLUSTAL W: improving the sensitivity of progressive multiple sequence alignment through sequence weighting, position-specific gap penalties and weight matrix choice. *Nucleic Acids Res*. 22, 4673 - 4680.

Zuckerkandl, E., and Pauling L. (1965). Evolutionary divergence and convergence in proteins. Edited in Evolving Genes and Proteins by V. Bryson and H.J. Vogel, pp. 97-166. Academic Press, New York.
